# Sex-specific association of rs4746172 of *VCL* gene with hypertension in two Han populations from Southern China

**DOI:** 10.1038/srep15245

**Published:** 2015-10-21

**Authors:** Qin Yu, Hong-Peng Sun, Wan-Qun Chen, Xiao-Qiong Chen, Yong Xu, Yong-Han He, Qing-Peng Kong

**Affiliations:** 1 State Key Laboratory of Genetic Resources and Evolution, Kunming Institute of Zoology, the Chinese Academy of Sciences, Kunming 650223, China; 2 Jiangsu Key Laboratory of Preventive and Translational Medicine for Geriatric Diseases, School of Public Health, Soochow University, Suzhou 215123, China; 3 Department of Biochemistry, Jinan University, Guangzhou 510632, China

## Abstract

Hypertension is the most common and lethal risk factor for cardiovascular disease (CVD). Numerous variants have been associated with hypertension, however, most of which failed to get replication due to ethnic differences. In this study, we analyzed associations of 10 newly reported single nucleotide polymorphisms (SNPs) in Europeans with hypertension in Chinese. A total of 1766 samples consisting of 880 subjects with hypertension and 886 controls were collected and the SNPs were genotyped using multiple assays based on the SNaPshot mini-sequencing approach. Our results revealed a significant genotypic association of rs4746172 of *VCL* with hypertension with a lower frequency of minor allele in male subjects (OR = 0.70, 95% CI: 0.54–0.92, p = 0.011) but not in females. To validate the result, we genotyped the SNPs in another Chinese population with 546 individuals, and got a consistent association for the rs4746172 (OR = 0.56, 95% CI: 0.38-0.82, p = 2.4 × 10^−3^) in males. The *VCL*-encoding protein was involved in cardiomyopathy that associated with hypertension, therefore our results suggest the rs4746172 of *VCL* may be a novel target for clinical interventions to reduce CVD risk by regulating blood pressure in male Chinese.

Hypertension (HT) is one of the most common diseases around the world with about 1 out of every 3 adults have HT in US^1^, and among Chinese adults, the prevalence of HT is 26.6% according to the 2007–2008 survey conducted by the China Diabetes and metabolic Disorders Study^2^. Moreover, HT is an important risk factor for cardiovascular diseases, such as heart failure (HF)^3^, stroke^4^ and myocardial infarction^5^. Much of the excess CVD risk led by HT can be ameliorated through blood pressure (BP)-lowering interventions. Accordingly, identifying novel genes and pathways involved in the regulation of BP may provide new ways of reducing BP and CVD risk. The causation of HT is complex, environmental (e.g. lifestyle) or genetic or combination of both could elevate BP^6^. Although unhealthy lifestyle, such as excess intake of salt and alcohol and lack of exercise, are shown to increase BP and the risk of developing HT^7^, a substantial contribution of genetic factors has been documented in many studies, in which numerous genes and single nucleotide polymorphisms (SNPs) have been shown involved in the regulation of BP or HT^8^,^9^,^10^.

With the development of the next generation sequence, the understanding of genetic component of BP or HT has made a big progress, especially in the genome-wide association studies (GWAS)^11^,^12^. However, many studies failed to get replication association in independent populations, which may be accounted by ethnic differences among populations^13^,^14^. Therefore, it’s a tendency and necessary to identify universal BP-associated loci using multiethnic samples^15^. Recently, novel associations between several SNPs and BP traits were identified in more than ten thousands of individuals of European ancestry^16^,^17^. Zhang and his colleagues used novel strategies to discover some novel BP loci, one of which was proved to be a new hypertension-susceptibility locus^18^. However, these candidate BP-associated loci have not been investigated in Asia populations. Accordingly, the study is designed to replicate the reported gene SNPs in Chinese cohorts, aiming to find candidate gene loci involved in BP regulation. As a result, we found that the *VCL* rs4746172 had a lower frequency of minor allele and associated with HT in male Chinese. Importantly, the result was well replicated in another smaller Chinese population. Therefore, the *VCL* rs4746172 may serve as a novel target for clinical interventions to lower BP and CVD risk in male Chinese.

## Methods

### Subjects

A total of 1766 subjects were recruited from Jiangsu province, China (hereafter referred to as Jiangsu population). Among them, there were 880 subjects with HT defined as systolic blood pressure (SBP) ≥140 mmHg and/or diastolic blood pressure (DBP) ≥90 mmHg according to the HT criterion^19^. Normal BP was defined as SBP <140 mmHg and DBP <90 mmHg. Clinical characteristics, including age, sex, SBP, DBP, height, body weight, triglyceride (TG), total cholesterol (TC), high density lipoprotein cholesterol (HDL-c), low density lipoprotein cholesterol (LDL-c) and fasting blood glucose (GLU) were collected. Pulse pressure (PP) was calculated as SBP minus DBP, and median arterial pressure (MAP) was defined as 1/3 SBP plus 2/3 DBP. Another population containing 546 individuals with 269 HT patients and 277 controls from Guangdong province of China was collected for use of validation (hereafter referred to as Guangdong population). The study protocol was approved by the Ethics Committee at Kunming Institute of Zoology, Chinese Academy of Sciences. Written informed consent was obtained from each of the participants. All of the methods were carried out in “accordance” with the approved guidelines (http://www.nature.com/srep/policies/index.html#experimental-subjects).

### SNP selection and genotyping

Ten recently reported SNPs associated with DBP, SBP, PP, MAP or HT in Europeans^16^,^17^,^18^ were selected for genotyping (Supplemental Table S1). Total genomic DNA was extracted from the whole blood using the AxyPrep^TM^ Blood Genomic DNA Miniprep Kit (Axygen, USA) according to the manufacturer’s protocol, and quantified spectrophotometrically by OD_260_/OD_280_ ratio and stored at 4 °C for short-term. Primers were optimally designed using a web-based software provided by Beckman Coulter (available at www.autoprimer.com) and were listed in Supplemental Table S2 and Table S3. Genotyping was performed based on the SNaPshot mini-sequencing approach which can detect multiple polymorphisms in a single assay^20^,^21^. The resulting data were analyzed using GeneMarker (SoftGenetics, State College, PA).

### Statistical analysis

Continuous variables were displayed as mean ± SD and differences between groups were assessed by student’s *t*-test or ANOVA analysis. The difference of categorical variables was assessed by the Chi-square test. Odds ratios (ORs) and 95% confidence intervals (CIs) of alleles were calculated by the R software using Fisher’s exact test (2-side). Odds ratios (ORs) and 95% confidence intervals (CIs) of genotypes were calculated using SNPStats adjusted by age and sex, under co-dominant, dominant, recessive, over-dominant and log-additive models^22^. For sex subgroup analysis, age was adjusted for the association of SNPs with HT.

## Results

### Clinical characteristics

Clinical characteristics of two populations used in this study were shown in Table 1. All subjects were the Han nationality. Age and sex distribution in Jiangsu population and age distribution in Guangdong population were different between the control and HT groups (p < 0.05). In Jiangsu population, SBP, DBP, PP, MAP, body weight, TG, TC, LDL-c and GLU were significantly higher in the HT group than that in control group (p < 0.05), however, there were not any differences in height and HDL-c between groups (p > 0.05). For Guangdong population, SBP, SBP, DBP, PP, MAP, weight, HDL-c and GLU were higher in the HT group in contrast to the controls (p < 0.05), with an increasing trend but no significance for height, TG, TC and LDL-c (p > 0.05).

### Allelic distributions and associations with HT

After genotyping, we compared the minor allele frequencies (MAFs) of the SNPs in Jiangsu and Guangdong populations to that of the Southern Han Chinese (CHS) from the 1000 Genomes Project. MAFs among 3 populations were quite similar (Supplemental Table S1). We then analyzed the association of the minor alleles of all SNPs with HT but found no significant associations in total Jiangsu population (p > 0.05, Table 2). However, the minor allele T of rs4746172 had a significant association with HT in male subjects of Jiangsu subjects (OR = 0.80, 95% CI = 0.65–0.97, p = 0.020) with a lower frequency (0.36) in HT group than that in the controls (0.41), however, no association was observed in females (OR = 1.11, 95% CI = 0.91–1.36, p = 0.298).

### Genotypic distributions and associations with HT

We next investigated the genotypic associations of all selected SNPs with HT in Jiangsu population. Genotypic distributions of all SNPs in the controls of Jiangsu population were in agreement with the Hardy–Weinberg Equilibrium (HWE) (all p > 0.05, Table 2). Odds ratios (ORs) were calculated using five genetic models (co-dominant, dominant, recessive, over-dominant and log-additive) adjusted by sex and age. As shown in Table 3, there were not any significant associations between the selected SNPs and HT in total Jiangsu samples (p > 0.05). Due to the known effect of sex on BP^23^, we divided the samples into male and female subgroups and then analyzed the associations of the SNPs with HT. Interestingly, we observed the rs59251428 was significantly associated with HT in the co-dominant and over-dominant models in males (OR = 1.37, 95% CI: 1.04–1.80, p = 0.035 and OR = 1.40, 95% CI: 1.08–1.83, p = 0.012, respectively). In addition, the rs4746172 were also found to associate with HT in either of co-dominant, dominant and additive models in males (OR = 0.71, 95% CI: 0.53–0.95, p = 0.039; OR = 0.70, 95% CI: 0.54–0.92, p = 0.011; and OR = 0.80, 95% CI: 0.66–0.97, p = 0.020, respectively), being consistent with its allelic associations described above.

### Association validation of SNPs with HT in another independent population

To confirm above associations of SNPs with HT in Jiangsu population, we further genotyped the SNPs in Guangdong samples (Supplemental Table S4). As shown in Table 4, we did not found any associations for the rs59251428 with HT in any of the five models. However, we did observe that the rs4746172 was associated with HT in Guangdong population (co-dominant, OR = 0.58, 95% CI: 0.40–0.84, p = 2.9 × 10^−3^; dominant, OR = 0.67, 95% CI: 0.47–0.95, p = 0.024; and over-dominant, OR = 0.55, 95% CI: 0.39–0.79, p = 9.0 × 10^−4^). Consistently, subgroup analysis by sex revealed the association of rs4746172 with HT were only in male subjects (co-dominant, OR = 0.58, 95% CI: 0.39–0.86, p = 8.6 × 10^−3^; dominant, OR = 0.66, 95% CI: 0.45–0.96, p = 0.028; and over-dominant, OR = 0.56, 95% CI: 0.38–0.82, p = 2.4 × 10^−3^, Table 4), but not in the females (co-dominant, OR = 0.63, 95% CI: 0.21–1.87, p = 0.290; dominant, OR = 0.83, 95% CI: 0.30–2.28, p = 0.720; and over-dominant, OR = 0.54, 95% CI: 0.19–1.53, p = 0.240, Table 4). In this population, a marginally significant association of rs2014408 with HT was observed (over-dominant, OR = 0.68, 95% CI: 0.48–0.98, p = 0.036, Table 4). However, the association disappeared in stratification analysis by sex (Table 4). There were not any associations for the rest SNPs with HT in Guangdong population.

### Association of SNPs with blood pressure parameters

Since there was an association between some of the selected SNPs and HT, we next wondered whether they were associated with BP parameters, including SBP, DBP, PP and MAP. For the rs4746172, we did see a decreased trend for SBP, DBP and MAP in CT or TT carriers compared to the CC subjects in male subject despite of no significance in Jiangsu samples (Supplemental Fig. S1). There were not any associations for the rest SNPs with BP parameters (Supplemental Table S5 and supplemental Table S6). In Guangdong samples, the association of rs4746172 with DBP were significant among 3 genotype carriers (p = 0.020, Supplemental Table S7). The results further supported the association of the rs4746172 with HT in male Chinese, which may be mediated by its association with DBP.

## Discussion

In this study, we investigated the associations of 10 newly reported BP-associated SNPs in Europeans with BP or HT in Chinese populations, and finally identified a lower allelic frequency of rs4746172 in HT subjects in male Chinese. This result was well replicated in another independent Chinese population. To our knowledge, this the first report on the male-specific association of rs4746172 with HT in two Chinese populations.

In Jiangsu samples, we just found two male-specific hypertension-related loci, rs59251428 and rs4746172, were associated with HT, while the remaining 8 loci were not. The failed replication of association for the rest 8 SNPs with HT compared to the European population was most likely due to the ethnic differences between both populations. For the rs59251428, we failed to replicate its association with HT between two Chinese populations, which may result from several reasons. Firstly, the sample size in the validation population might be relatively small to test the significance. Secondly, geographic and social difference may affect the results of replication. The third is the average age between two populations. Guangdong subjects are younger than Jiangsu population consisting of old subjects which are prone to develop HT as documented in other study^24^. Even though, the association of rs4746172 with HT in Jiangsu samples was validated in Guangdong population with smaller sample size. Of notice is that the minor allelic frequency was lower in HT subjects than in the controls. Although it was reported to be a risk locus for BP, its lower allelic frequency and OR value indicate a likely protective role in developing HT. The strong association with BP in Europeans and association with HT in Chinese suggest its great potential of being a influential factor for HT in both populations.

The rs4746172 is located in the gene *VCL* (vinculin) encoding a cytoskeletal protein that is associated with the maintenance of cell-to-cell and cell to matric junctions and plays a crucial role in normal embryonic development and cardiac function^25^. Mutation, altered expression and location of *VCL* have been associated with cardiomyopathy^26^,^27^,^28^,^29^, which emphasize the importance of *VCL* in human heart. Moreover, it has been suggested that the subjects harbored mutations of usually suffer from increased heart workload^25^, which may promote the development of HT^30^. Indeed, several studies have reported that the patients with dilated cardiomyopathy had a higher prevalence of HT than the general population^31^. So it was plausible that the rs4746172 led to increased heart workload, and indirectly caused HT. Until now, there has been no direct evidence on the relationship between *VCL* gene and HT. Consistent with the case that the SNP rs4746172 was associated with DBP in Europeans^17^, we also observed its association with DBP in Guangdong individuals. That the decreased trend for BP parameters in CT or TT carriers than the wild genotype (CC) carriers but with no significance is most likely due to the protective role of lower allelic frequency in HT patients, which, however, did not affect the association of rs4746172 with HT. It was worthy pointing out that the rs4746172-HT association was sex-specific, existed only in male but not in female Chinese. In fact, the sex differences in the regulation of BP had been well studied in the past decades. Epidemiological studies indicate that men have higher BP than age-matched, premenopausal women^23^,^32^. There were also some studies reporting sex differences in association between genetic factors and HT^33^,^34^. However, the mechanism of sex on BP is not fully understood. One possible mechanism may be the role of sex hormones. Solid evidence has demonstrated that increases in androgens or losses of estrogens, or even increased ratio of androgens/estrogens can promote higher BP^35^. In addition, the sex-specific difference in genotype-phenotype associations may be due to sex-specific genetic architecture. The autosomal genome is shared by both the male and female, but the gene expression is sexually dimorphic^36^,^37^. Furthermore, sex is considered as an “environmental” variable and gene expression may be different via interaction with sex, and the genotype-sex interaction effects on many human diseases and traits are common, such as HT and BP^38^.

The association of rs4746172 with HT in this study was convincingly supported by the replication in two independent populations as well as the reported result in Europeans. However, several limitations should be acknowledged. One is that the sample sizes of both populations are not equivalent, especially for Guangdong population. Another is the population characteristics with bias, such as the ratio of the male and female, and age of samples. Third, despite its association with blood pressure in Caucasians and Chinese, the rs4746172 is a tag SNP and therefore its roles in the regulation of blood pressure deserve further studies in the future. In addition, the causation of HT is the complex multifactorial interplay of genetic and environmental factor, but here we focused on genetic factors without taking much account into some environmental factors associated with HT except for sex, such as salt and alcohol intake, and physical activities.

In conclusion, our results suggest the rs4746172 of *VCL* was associated with HT in male subjects with a lower frequency of risk allele, which was validated in another Chinese population. The lower frequency of rs4746172 may be a male-specific protective factor for cardiovascular diseases, which may serve as a novel target and provide strategies for clinical interventions to reduce CVD risk in male Chinese.

## Additional Information

**How to cite this article**: Yu, Q. *et al*. Sex-specific association of rs4746172 of *VCL* gene with hypertension in two Han populations from Southern China. *Sci. Rep*. **5**, 15245; doi: 10.1038/srep15245 (2015).

## Supplementary Material

Supplementary Information

## Figures and Tables

**Table 1:** Clinical characteristics of control and HT groups in two Chinese populations.

Variables	Jiangsu population (n = 1766)			Guangdong population (n = 546)		
	Control (n = 886)	HT (n = 880)	p	Control (n = 277)	HT (n = 269)	p
Age, years	70.46 ± 6.50	71.11 ± 6.06	0.030	42.60 ± 10.16	47.64 ± 11.75	1.34 × 10^−7^
Sex (M/F)	479/407	419/461	**7.74× 10**^**−3**^	237/40	234/35	0.718
SBP, mmHg	126.78 ± 9.15	163.18 ± 16.15	**<2.20 × 10**^**−16**^	117.12 ± 10.57	141.07 ± 18.11	**<2.20 × 10**^**−16**^
DBP, mmHg	76.80 ± 8.16	98.46 ± 7.82	**<2.20 × × 10**^**−16**^	76.44 ± 6.97	93.88 ± 8.84	**<2.20 × 10**^**−16**^
PP, mmHg	49.98 ± 9.73	64.72 ± 15.11	**<2.20 × 10**^**−16**^	40.68 ± 8.23	47.19 ± 16.87	**6.32 × 10**^**-6**^
MAP, mmHg	93.46 ± 7.16	120.03 ± 8.78	**<2.20 × 10**^**−16**^	90.00 ± 7.38	109.61 ± 9.91	**<2.20 × 10**^**−16**^
Height, cm	158.89 ± 8.62	158.33 ± 8.70	0.197	166.53 ± 6.61	167.08 ± 6.97	0.349
Weight, kg	56.70 ± 9.43	59.30 ± 10.41	**2.37 × 10**^**−7**^	68.11 ± 9.27	73.87 ± 12.51	**2.48 × 10**^**−9**^
TG, mmol/L	1.32 ± 0.68	1.46 ± 0.79	**8.58 × 10**^**−5**^	2.24 ± 1.28	2.36 ± 1.80	0.113
TC, mmol/L	5.05 ± 0.86	5.29 ± 0.97	**2.78 × 10**^**−8**^	5.58 ± 1.23	5.59 ± 0.99	0.908
HDL-c, mmol/L	1.55 ± 0.42	1.59 ± 0.42	0.107	1.21 ± 0.28	1.30 ± 0.30	**4.08 × 10**^**−4**^
LDL-c, mmol/L	2.75 ± 0.70	2.91 ± 0.78	**6.34 × 10**^**−6**^	3.24 ± 0.85	3.32 ± 0.70	0.264
GLU, mmol/L	5.77 ± 0.99	6.16 ± 1.23	**7.87 × 10**^**−16**^	5.40 ± 1.60	5.79 ± 1.81	**7.05 × 10**^**−4**^

M, male; F, female; SBP, systolic blood pressure; DBP, diastolic blood pressure; PP, pulse pressure; MAP, median arterial pressure; TG, triglyceride; TC, total cholesterol; HDL-c, high density lipoprotein cholesterol; LDL-c, low density lipoprotein cholesterol; GLU, fasting blood glucose; HT, hypertensive patient. PP was calculated as SBP minus DBP, and MAP was calculated as 1/3 SBP plus 2/3 DBP. All quantitative data were presented as mean ± SD. P values < 0.05 were marked in bold.

**Table 2:** Allelic ORs and 95% CI for HT in Jiangsu population.

SNP	Allele	HWE	All				Female				Male			
			Control n (freq)	HT n (freq)	OR (95% CI)	p	Control n (freq)	HT n (freq)	OR (95% CI)	p	Control n (freq)	HT n (freq)	OR (95% CI)	p
rs16823124	G	1.00	1015 (0.57)	1008 (0.57)	1.00 (0.87–1.15)	1.000	470 (0.58)	536 (0.58)	0.98 (0.81–1.20)	0.884	545 (0.57)	472 (0.56)	1.02 (0.84–1.24)	0.812
	A		757 (0.43)	752 (0.43)			344 (0.42)	386 (0.42)			413 (0.43)	366 (0.44)		
rs4245739	A	0.49	1723 (0.97)	1720 (0.98)	0.82 (0.52–1.28)	0.391	790 (0.97)	905 (0.98)	0.62 (0.31–1.21)	0.154	933 (0.97)	815 (0.97)	1.05 (0.57–1.95)	0.884
	C		49 (0.03)	40 (0.02)			24 (0.03)	17 (0.02)			25 (0.03)	23 (0.03)		
rs2158394	C	0.06	1156 (0.65)	1128 (0.64)	1.05 (0.92–1.21)	0.459	518 (0.64)	577 (0.63)	1.05 (0.86–1.29)	0.617	638 (0.67)	551 (0.66)	1.04 (0.85–1.27)	0.726
	G		612 (0.35)	630 (0.36)			292 (0.36)	343 (0.37)			320 (0.33)	287 (0.34)		
rs1570105	A	0.73	1001 (0.56)	1011 (0.57)	0.96 (0.84–1.10)	0.587	458 (0.56)	532 (0.58)	0.94 (0.78–1.15)	0.560	543 (0.57)	479 (0.57)	0.98 (0.81–1.19)	0.849
	G		771 (0.44)	749 (0.43)			356 (0.44)	390 (0.42)			415 (0.43)	359 (0.43)		
rs2014408	C	0.63	1256 (0.71)	1270 (0.72)	0.94 (0.81–1.09)	0.412	585 (0.72)	676 (0.73)	0.93 (0.75–1.16)	0.518	671 (0.70)	594 (0.71)	0.96 (0.78–1.18)	0.717
	T		516 (0.29)	490 (0.28)			229 (0.28)	246 (0.27)			287 (0.30)	244 (0.29)		
rs33063	G	0.17	1657 (0.94)	1653 (0.94)	0.93 (0.70–1.24)	0.628	756 (0.93)	870 (0.94)	0.78 (0.52–1.17)	0.236	901 (0.94)	783 (0.93)	1.11 (0.74–1.66)	0.625
	A		115 (0.06)	107 (0.06)			58 (0.07)	52 (0.06)			57 (0.06)	55 (0.07)		
rs7297416	C	0.08	1091 (0.62)	1071 (0.61)	1.03 (0.9–1.18)	0.679	503 (0.62)	559 (0.61)	1.05 (0.86–1.28)	0.622	588 (0.61)	512 (0.61)	1.01 (0.83–1.23)	0.923
	A		681 (0.38)	689 (0.39)			311 (0.38)	363 (0.39)			370 (0.39)	326 (0.39)		
rs59251428	G	0.40	1279 (0.72)	1265 (0.72)	1.02 (0.87–1.18)	0.851	582 (0.71)	673 (0.73)	0.93 (0.75–1.15)	0.519	697 (0.73)	592 (0.71)	1.11 (0.90–1.37)	0.344
	T		493 (0.28)	495 (0.28)			232 (0.29)	249 (0.27)			261 (0.27)	246 (0.29)		
rs3858313	A	0.52	1100 (0.62)	1124 (0.64)	0.93 (0.81–1.06)	0.280	514 (0.63)	586 (0.64)	0.98 (0.80–1.20)	0.881	586 (0.61)	538 (0.64)	0.88 (0.72–1.07)	0.187
	G		672 (0.38)	636 (0.36)			300 (0.37)	336 (0.36)			372 (0.39)	300 (0.36)		
rs4746172	C	0.44	1077 (0.61)	1097 (0.62)	0.94 (0.82–1.08)	0.350	515 (0.63)	560 (0.61)	1.11 (0.91–1.36)	0.298	562 (0.59)	537 (0.64)	**0.80** (**0.65–0.97)**	**0.020**
	T		695 (0.39)	663 (0.38)			299 (0.37)	362 (0.39)			396 (0.41)	301 (0.36)		

HWE, Hardy–Weinberg Equilibrium, p wwvalue for HWE in control was shown; HT, hypertensive patient; OR, Odds ratio; 95% CI, 95% confidence interval; freq means the frequencies of allele. P values < 0.05 were marked in bold.

**Table 3:** Genotypic associations of SNPs with HT in Jiangsu population.

SNP	Sex	OR (95% CI)									
		Co-dominant	p	Dominant	p	Recessive	p	Over-dominant	p	Log-additive	p
rs16823124	all	0.97 (0.79–1.20) 1.02 (0.77–1.33)	0.940	0.98 (0.81–1.20)	0.880	1.03 (0.81–1.31)	0.800	0.97 (0.80–1.17)	0.730	1.00 (0.88–1.15)	0.970
	female	0.85 (0.63–1.15) 1.01 (0.68–1.51)	0.490	0.89 (0.67–1.18)	0.420	1.11 (0.78–1.59)	0.560	0.85 (0.65–1.11)	0.230	0.98 (0.80–1.19)	0.810
	male	1.10 (0.82–1.48) 1.03 (0.71–1.49)	0.810	1.08 (0.81–1.42)	0.610	0.97 (0.70–1.35)	0.860	1.09 (0.84–1.42)	0.530	1.02 (0.85–1.23)	0.810
rs4245739	all	0.85 (0.55–1.31) 0.00 (0.00–NA)	0.410	0.83 (0.54–1.28)	0.400	0.00 (0.00–NA)	0.270	0.85 (0.55–1.31)	0.460	0.82 (0.54–1.25)	0.360
	female	0.61 (0.32–1.16) NA	0.130								
	male	1.15 (0.63–2.08) 0.00 (0.00–NA)	0.480	1.10 (0.61–1.98)	0.750	0.00 (0.00–NA)	0.260	1.15 (0.63–2.08)	0.640	1.05 (0.59–1.86)	0.860
rs2158394	all	1.08 (0.88-1.31) 1.08 (0.79–1.50)	0.750	1.08 (0.89–1.30)	0.440	1.04 (0.77–1.41)	0.790	1.06 (0.88–1.28)	0.560	1.05 (0.91–1.22)	0.480
	female	1.12 (0.84–1.49) 1.11 (0.71–1.73)	0.750	1.11 (0.84–1.47)	0.450	1.04 (0.69–1.58)	0.850	1.09 (0.83–1.42)	0.530	1.07 (0.87–1.31)	0.510
	male	1.05 (0.79–1.38) 1.08 (0.68–1.71)	0.920	1.05 (0.81–1.37)	0.700	1.05 (0.68–1.63)	0.830	1.03 (0.80–1.34)	0.800	1.04 (0.85–1.28)	0.700
rs1570105	all	0.87 (0.71–1.08) 0.93 (0.71–1.23)	0.450	0.89 (0.73–1.09)	0.250	1.01 (0.80–1.29)	0.910	0.89 (0.74–1.08)	0.250	0.95 (0.83–1.09)	0.480
	female	0.92 (0.68–1.25) 0.86 (0.59–1.28)	0.750	0.91 (0.68–1.21)	0.510	0.91 (0.64–1.28)	0.580	0.97 (0.75–1.27)	0.850	0.93 (0.77–1.12)	0.450
	male	0.83 (0.62–1.12) 1.01 (0.69–1.47)	0.360	0.88 (0.66–1.16)	0.360	1.13 (0.81–1.58)	0.480	0.83 (0.64–1.08)	0.160	0.98 (0.81–1.18)	0.830
rs2014408	all	1.04 (0.85–1.26) 0.76 (0.53–1.10)	0.270	0.99 (0.82–1.19)	0.890	0.75 (0.53–1.07)	0.110	1.07 (0.89–1.30)	0.470	0.94 (0.81–1.09)	0.430
	female	1.03 (0.78–1.36) 0.74 (0.44–1.24)	0.460	0.98 (0.75–1.28)	0.860	0.73 (0.44–1.21)	0.220	1.07 (0.81–1.41)	0.630	0.93 (0.75–1.15)	0.520
	male	1.05 (0.80–1.38) 0.80 (0.48–1.32)	0.570	1.00 (0.77–1.31)	0.970	0.78 (0.48–1.27)	0.320	1.08 (0.83–1.41)	0.560	0.96 (0.78–1.18)	0.690
rs33063	all	0.89 (0.67–1.19) 2.16 (0.19–23.92)	0.600	0.91 (0.68–1.20)	0.490	2.19 (0.20–24.24)	0.510	0.89 (0.67–1.19)	0.440	0.92 (0.70–1.21)	0.550
	female	0.74 (0.49–1.10) NA	0.140								
	male	1.07 (0.71–1.61) 2.30 (0.21–25.47)	0.740	1.09 (0.73–1.63)	0.660	2.28 (0.21–25.25)	0.490	1.07 (0.71–1.60)	0.750	1.11 (0.76–1.63)	0.590
rs7297416	all	1.07 (0.88–1.32) 1.04 (0.77–1.40)	0.790	1.07 (0.88–1.30)	0.520	0.99 (0.76–1.31)	0.970	1.06 (0.88–1.28)	0.520	1.03 (0.90–1.19)	0.660
	female	1.12 (0.83–1.49) 1.07 (0.70–1.61)	0.770	1.10 (0.84–1.46)	0.490	1.00 (0.68–1.46)	0.990	1.10 (0.84–1.43)	0.500	1.05 (0.86–1.28)	0.620
	male	1.04 (0.78-1.38) 1.01 (0.65–1.56)	0.970	1.03 (0.78–1.36)	0.830	0.99 (0.66–1.47)	0.950	1.03 (0.80–1.35)	0.800	1.01 (0.83–1.24)	0.900
rs59251428	all	1.06 (0.87–1.29) 0.98 (0.67–1.43)	0.800	1.05 (0.87–1.27)	0.600	0.95 (0.66–1.38)	0.800	1.07 (0.88–1.29)	0.510	1.02 (0.88–1.19)	0.750
	female	0.81 (0.61–1.08) 1.15 (0.67–1.95)	0.240	0.86 (0.65–1.12)	0.260	1.26 (0.75–2.11)	0.380	0.80 (0.61–1.05)	0.110	0.94 (0.76–1.17)	0.590
	male	**1.37 (1.04**–**1.80)** 0.83 (0.48–1.44)	**0.035**	1.28 (0.99–1.67)	0.061	0.71 (0.42–1.23)	0.220	**1.40 (1.08**–**1.83)**	**0.012**	1.12 (0.90–1.38)	0.300
rs3858313	all	0.92 (0.75–1.13) 0.86 (0.64–1.15)	0.540	0.91 (0.75–1.10)	0.320	0.90 (0.69–1.17)	0.430	0.96 (0.80–1.16)	0.670	0.93 (0.81–1.06)	0.270
	female	1.04 (0.78–1.39) 0.92 (0.61–1.39)	0.820	1.01 (0.77–1.33)	0.930	0.90 (0.61–1.32)	0.580	1.07 (0.82–1.40)	0.640	0.98 (0.81–1.19)	0.830
	male	0.82 (0.61–1.08) 0.81 (0.54–1.22)	0.330	0.82 (0.62–1.07)	0.140	0.91 (0.62–1.32)	0.610	0.86 (0.66–1.12)	0.270	0.88 (0.73–1.06)	0.190
rs4746172	all	0.96 (0.79–1.18) 0.86 (0.65–1.15)	0.590	0.94 (0.77–1.14)	0.510	0.88 (0.68–1.14)	0.340	1.00 (0.83–1.21)	0.960	0.94 (0.82–1.07)	0.340
	female	1.31 (0.98–1.75) 1.11 (0.74–1.67)	0.190	1.26 (0.95–1.65)	0.100	0.96 (0.66–1.40)	0.830	1.27 (0.97–1.66)	0.081	1.11 (0.91–1.35)	0.300
	male	**0.71 (0.53**–**0.95)** 0.68 (0.46–1.01)	**0.039**	**0.70 (0.54**–**0.92)**	**0.011**	0.82 (0.57–1.18)	0.280	0.80 (0.61–1.04)	0.091	**0.80 (0.66**–**0.97)**	**0.020**

OR, Odds ratio; 95% CI, 95% confidence interval. For total samples, p values were adjusted by sex and age, and for sex subgroup analyses, p values were adjusted by age. P values < 0.05 were marked in bold.

**Table 4:** Genotypic association of SNPs with HT in Guangdong population.

SNP	Sex	OR (95% CI)									
		Co-dominant	p	Dominant	p	Recessive	p	Over-dominant	p	Log-additive	p
rs16823124	all	0.71 (0.48–1.04) 0.66 (0.39–1.11)	0.140	0.69 (0.48–1.00)	0.048	0.81 (0.51–1.29)	0.370	0.80 (0.57–1.14)	0.220	0.79 (0.62–1.02)	0.066
	female	0.54 (0.17–1.66) 0.31 (0.06–1.68)	0.330	0.48 (0.16–1.43)	0.180	0.46 (0.10–2.11)	0.310	0.75 (0.27–2.08)	0.590	0.55 (0.25–1.22)	0.140
	male	0.74 (0.49–1.11) 0.72 (0.42–1.24)	0.290	0.73 (0.50–1.08)	0.120	0.86 (0.53–1.40)	0.540	0.82 (0.57–1.19)	0.300	0.83 (0.64–1.08)	0.160
rs2158394	all	1.08 (0.74–1.57) 1.49 (0.87–2.56)	0.350	1.17 (0.82–1.66)	0.400	1.43 (0.86–2.37)	0.160	0.98 (0.69–1.38)	0.890	1.18 (0.92–1.52)	0.190
	female	1.57 (0.49–5.07) 4.10 (0.81–20.83)	0.220	1.98 (0.66–5.97)	0.220	3.15 (0.72–13.70)	0.110	0.99 (0.35–2.79)	0.990	1.93 (0.89–4.18)	0.091
	male	1.02 (0.68–1.52) 1.29 (0.73–2.31)	0.660	1.08 (0.74–1.57)	0.700	1.28 (0.75–2.20)	0.360	0.95 (0.66–1.39)	0.810	1.11 (0.85–1.45)	0.460
rs1570105	all	0.91 (0.61–1.35) 1.36 (0.82–2.26)	0.240	1.01 (0.70–1.47)	0.940	1.44 (0.93–2.25)	0.100	0.81 (0.57–1.14)	0.230	1.13 (0.88–1.45)	0.340
	female	0.42 (0.13–1.42) 0.55 (0.12–2.53)	0.360	0.46 (0.15–1.42)	0.170	0.90 (0.24–3.40)	0.870	0.52 (0.18–1.54)	0.230	0.68 (0.32–1.44)	0.310
	male	0.97 (0.64–1.48) 1.52 (0.88–2.61)	0.200	1.10 (0.74–1.64)	0.640	1.54 (0.96–2.48)	0.072	0.83 (0.57–1.20)	0.330	1.20 (0.92–1.56)	0.190
rs2014408	all	0.70 (0.49–1.01) 1.38 (0.64–3.02)	0.078	0.77 (0.54–1.09)	0.130	1.60 (0.75–3.44)	0.220	0.68 (0.48–0.98)	0.036	0.89 (0.67–1.19)	0.440
	female	0.73 (0.26–2.06) NA (0.00–NA)	0.200	0.85 (0.31–2.34)	0.760	NA (0.00–NA)	0.089	0.65 (0.23–1.80)	0.410	1.09 (0.44–2.68)	0.850
	male	0.71 (0.48–1.04) 1.22 (0.55–2.70)	0.150	0.76 (0.52–1.10)	0.150	1.40 (0.64–3.06)	0.390	0.69 (0.47–1.02)	0.059	0.88 (0.65–1.19)	0.400
rs33063	all	0.66 (0.38–1.13) 0.22 (0.02–2.18)	0.120	0.62 (0.36–1.05)	0.073	0.23 (0.02–2.30)	0.170	0.67 (0.39–1.15)	0.140	0.62 (0.38–1.00)	0.049
	female	1.20 (0.27–5.36) 0.00 (0.00–NA)	0.420	0.91 (0.22–3.77)	0.900	0.00 (0.00–NA)	0.190	1.25 (0.28–5.56)	0.770	0.75 (0.22–2.56)	0.640
	male	0.60 (0.34–1.08) 0.32 (0.03–3.77)	0.160	0.58 (0.33–1.03)	0.062	0.35 (0.03–4.02)	0.380	0.61 (0.34–1.09)	0.091	0.60 (0.35–1.02)	0.054
rs7297416	all	1.10 (0.75–1.61) 1.18 (0.69–2.03)	0.810	1.12 (0.78–1.60)	0.560	1.12 (0.68–1.83)	0.650	1.04 (0.74–1.48)	0.810	1.09 (0.84–1.41)	0.520
	female	0.89 (0.30–2.63) 0.81 (0.16–4.06)	0.960	0.87 (0.32–2.42)	0.800	0.86 (0.19–3.91)	0.850	0.94 (0.34–2.59)	0.900	0.90 (0.43–1.88)	0.780
	male	1.14 (0.76–1.72) 1.24 (0.70–2.21)	0.720	1.16 (0.79–1.72)	0.450	1.15 (0.68–1.93)	0.610	1.07 (0.74–1.55)	0.720	1.12 (0.85–1.47)	0.420
rs59251428	all	0.95 (0.66–1.37) 1.13 (0.63–2.01)	0.840	0.98 (0.70–1.39)	0.930	1.16 (0.67–2.01)	0.600	0.93 (0.65–1.32)	0.680	1.02 (0.79–1.32)	0.860
	female	0.45 (0.15–1.40) 0.26 (0.05–1.37)	0.180	0.40 (0.14–1.16)	0.085	0.39 (0.08–1.82)	0.230	0.63 (0.22–1.75)	0.370	0.49 (0.23–1.07)	0.066
	male	1.04 (0.71–1.54) 1.36 (0.73–2.55)	0.620	1.10 (0.76–1.60)	0.810	1.33 (0.73–2.43)	0.340	0.99 (0.68–1.43)	0.940	1.12 (0.85–1.48)	0.410
rs3858313	all	1.29 (0.88–1.89) 1.06 (0.64–1.77)	0.400	1.22 (0.86–1.74)	0.260	0.93 (0.58–1.49)	0.760	1.27 (0.89–1.80)	0.180	1.08 (0.85–1.38)	0.540
	female	1.34 (0.45–3.99) 1.36 (0.26–7.13)	0.850	1.35 (0.48–3.75)	0.570	1.18 (0.24–5.69)	0.840	1.26 (0.45–3.55)	0.660	1.22 (0.58–2.57)	0.610
	male	1.29 (0.86–1.94) 1.05 (0.61–1.79)	0.440	1.22 (0.83–1.77)	0.310	0.92 (0.56–1.50)	0.730	1.27 (0.88–1.85)	0.210	1.07 (0.83–1.38)	0.610
rs4746172	all	0.58 (0.40–0.84) 1.30 (0.70–2.43)	2.9 × 10^**−**3^	**0.67 (0.47**–**0.95)**	**0.024**	1.72 (0.95–3.12)	0.070	**0.55 (0.39**–**0.79)**	**9.0 × 10**^**−4**^	0.89 (0.68–1.16)	0.380
	female	0.63 (0.21–1.87) 2.70 (0.41–17.73)	0.290	0.83 (0.30–2.28)	0.720	3.34 (0.54–20.70)	0.180	0.54 (0.19–1.53)	0.240	1.14 (0.53–2.46)	0.740
	male	**0.58 (0.39**–**0.86)** 1.19 (0.61–2.33)	**8.6 × 10**^**−**3^	**0.66 (0.45**–**0.96)**	**0.028**	1.59 (0.85–3.00)	0.150	**0.56 (0.38**–**0.82)**	**2.4 × 10**^**−3**^	0.86 (0.65–1.15)	0.310

OR, Odds ratio; 95% CI, 95% confidence interval. For total samples, p values were adjusted by sex and age, and for sex subgroup analyses, p values were adjusted by age. P values < 0.05 were marked in bold.
