# Supplementary material for: Sex-specific association of rs4746172 of VCL gene with hypertension in two Han populations from Southern China
Source: Sci Rep. 2015 Oct 21;5:15245. doi: 10.1038/srep15245 (PMC4613695; doi:10.1038/srep15245)
Supplement: Supplementary Information [file srep15245-s1.pdf]

Supplemental materials

Sex-specific association of rs4746172 of *VCL* gene with hypertension in two Han populations from Southern China

Qin Yu, Hong-Peng Sun, Wan-Qun Chen, Xiao-Qiong Chen, Yong Xu, Yong-Han He, Qing-Peng Kong

Supplemental Table S1. Selected SNPs associated with blood pressure or hypertension.

SNP	Allele	MAF[a]	MAF[b]	MAF[c]	Type	Position (GRCh38)	Nearest gene	Reported association
rs2014408	T/C	0.27	0.28	0.26	intron	11: 16,343,736	*SOX6*	SBP
rs16823124	A/G	0.35	0.43	0.41	intron	2: 182359400	*PDE1A*	DBP, MAP
rs4746172	T/C	0.36	0.38	0.33	intron	10: 74096084	*VCL*	DBP, MAP
rs33063	A/G	0.08	0.06	0.07	intron	16: 69606314	*NFAT5*	PP
rs7297416	A/C	0.36	0.39	0.39	intron	12: 54049306	*HOXC4*	SBP
rs59251428	T/G	0.30	0.28	0.32	intron	3: 11247993	*HRH1*	SBP
rs2158394	G/C	0.30	0.35	0.37	2KB upstream variant	11: 1998765	*H19*	SBP, MAP
rs4245739	C/A	0.04	0.03	0.03	3 prime UTR variant	1: 204549714	*MDM4*	DBP
rs3858313	G/A	0.29	0.37	0.38	intron	10: 126232158	*ADAM12*	hypertension
rs1570105	G/A	0.42	0.43	0.44	intron	1: 234293981	*SLC35F3*	hypertension

MAF, minor allele frequency

[a]MAFs of SNPs in the Southern Han Chinese (CHS) of the 1000 Genomes Project.

[b]MAFs of SNPs in control of Jiangsu population.

[c]MAFs of SNPs in control of Guangdong population.

SBP, systolic blood pressure; DBP, diastolic blood pressure; PP, pulse pressure; MAP, median arterial pressure.

Supplemental Table S2. Amplification primers of ten SNPs.

SNP	Multiple PCR Primers 5’ → 3’	
	**Forward**	**Reverse**
rs16823124	AATAAATAACAGTAATAAATCCCTCCAT	TTGTAAATTTGTCCTCTCTAAAAGC
rs4245739	TGGTTATTAAGGTTTTTATAGCATAATG	ATTCTCTGACAGGTTGGAAATAAA
rs2158394	CTCAAGTCTCGAGTGTCAAAGC	AACAGAGGGTTTGCCGAA
rs1570105	AATTGGGGTCATCTTCGTCT	TTATGATTTATGCTTTTAGAAAACAGC
rs2014408	TAAATAGAAAATTATCTTCAAATGGGG	ACCAGAAGACTGTTGATGAAGTAGA
rs33063	ATATCTTGCAAACCCTAGTGTAGAG	AATACTAAATCCACAAGGAAAACTG
rs7297416	GAAGAGACAAATTTCAATGGAGTT	AGTGACCTGCATTTCACCTC
rs59251428	AATATTGAGGTCATAATTCCTTTCC	CCTCCTTATCAAATAAATGAGTTAGC
rs3858313	AAAGATGTACACATTCTAATTCCTGG	TTATAAGGACCTTTGTGGTTACGT
rs4746172	TAGAGCTTATTAGGAAAAAGCTTGC	AAGTCCATTTTAAGAAAAATATTGGC

Supplemental Table S3. Extension primers of ten SNPs.

SNP	Extension primers 5’ → 3’
rs16823124	CGATTCTTGATAGGTTTTATTGAGC
rs4245739	actgactgactgactgact TACGAACATAAAAATGCATTTATTC
rs2158394	gactgactgactgactgactgact ACCCAGAGTTCACGGGTCCAATCAC
rs1570105	tgactgactgactgactgactgactgact CATTTGATGCTGAATTCCACAAGTC
rs2014408	tgactgactgactgactgactgactgactgact TTCAGAGGGAAGTTTAGAAATTACA
rs33063	tgactgactgactgactgactgactgactgactgact GGCACAGACAGCATAATATCAATTA
rs7297416	tgactgactgactgactgactgactgactgactgactgact CCCATCAATAACCCCATGGCAGTGA
rs59251428	tgactgactgactgactgactgactgactgactgactgactgact TTTCCCGAACCATTTCTCCATGAGG
rs3858313	tgactgactgactgactgactgactgactgactgactgactgactgact AAAAGGACTTTGCTGATGTGATTAC
rs4746172	tgactgactgactgactgactgactgactgactgactgactgactgactgact AATTCCACTCAGTCAAGAGCCCTTT

Supplemental Table S4. Allelic associations of SNPs with HT in Guangdong population.

SNP	Allele	All				Female				Male			
		Control n (freq)	HT n (freq)	OR (95% CI)	p	Control n (freq)	HT n (freq)	OR (95% CI)	p	Control n (freq)	HT n (freq)	OR (95% CI)	p
rs16823124	G	313 (0.56)	334 (0.62)	0.79	0.065	44 (0.55)	47 (0.67)	0.60	0.136	269 (0.57)	287 (0.61)	0.83	0.164
	A	241 (0.44)	204 (0.38)	(0.62-1.02)		36 (0.45)	23 (0.33)	(0.29-1.22)		205 (0.43)	181 (0.39)	(0.63-1.08)	
rs4245739	A	541 (0.98)	510 (0.97)	1.31	0.573	78 (0.98)	69 (0.99)	NA		463 (0.98)	441 (0.97)	1.43	0.429
	C	13 (0.02)	16 (0.03)	(0.58-2.98)		2 (0.02)	1 (0.01)			11 (0.02)	15 (0.03)	(0.61-3.49)	
rs2158394	C	360 (0.65)	325 (0.61)	1.18	0.186	53 (0.66)	36 (0.51)	1.85	0.070	307 (0.65)	289 (0.63)	1.1	0．496
	G	192 (0.35)	205 (0.39)	(0.92-1.53)		27 (0.34)	34 (0.49)	(0.91-3.78)		165 (0.35)	171 (0.37)	(0.83-1.45)	
rs1570105	A	319 (0.58)	294 (0.55)	1.13	0.330	45 (0.56)	42 (0.60)	0.86	0.740	274 (0.58)	252 (0.54)	1.17	0.238
	G	235 (0.42)	244 (0.45)	(0.88-1.44)		35 (0.44)	28 (0.40)	(0.42-1.73)		200 (0.42)	216 (0.46)	(0.90-1.53)	
rs2014408	C	409 (0.74)	404 (0.75)	0.94	0.677	59 (0.74)	51 (0.73)	1.05	1.000	350 (0.74)	353 (0.75)	0.92	0.601
	T	145 (0.26)	134 (0.25)	(0.71-1.24)		21 (0.26)	19 (0.27)	(0.47-2.3)		124 (0.26)	115 (0.25)	(0.68-1.25)	
rs33063	G	509 (0.92)	501 (0.95)	0.65	0.092	73 (0.91)	65 (0.93)	0.80	0.771	436 (0.92)	436 (0.95)	0.63	0.089
	A	45 (0.08)	29 (0.05)	(0.39-1.09)		7 (0.09)	5 (0.07)	(0.19-3.10)		38 (0.08)	24 (0.05)	(0.36-1.1)	
rs7297416	C	339 (0.61)	322 (0.60)	1.06	0.665	51 (0.64)	47 (0.67)	0.86	0.732	288 (0.61)	275 (0.59)	1.09	0.550
	A	215 (0.39)	216 (0.40)	(0.82-1.36)		29 (0.36)	23 (0.33)	(0.41-1.78)		186 (0.39)	193 (0.41)	(0.83-1.42)	
rs59251428	G	379 (0.68)	363 (0.67)	1.04	0.746	48 (0.60)	49 (0.70)	0.64	0.233	331 (0.70)	314 (0.67)	1.14	0.400
	T	175 (0.32)	175 (0.33)	(0.80-1.36)		32 (0.40)	21 (0.30)	(0.31-1.34)		143 (0.30)	154 (0.33)	(0.85-1.51)	
rs3858313	A	347 (0.63)	332 (0.62)	1.04	0.755	54 (0.68)	47 (0.67)	1.02	1.000	293 (0.62)	285 (0.61)	1.04	0.789
	G	207 (0.37)	206 (0.38)	(0.81-1.34)		26 (0.32)	23 (0.33)	(0.48-2.13)		181 (0.38)	183 (0.39)	(0.79-1.36)	
rs4746172	C	362 (0.65)	359 (0.68)	0.90	0.440	55 (0.69)	49 (0.70)	0.94	1.000	307 (0.65)	310 (0.67)	0.89	0.408
	T	192 (0.35)	171 (0.32)	(0.69-1.17)		25 (0.31)	21 (0.30)	(0.44-2.00)		167 (0.35)	150 (0.33)	(0.67-1.18)	

HT, hypertensive patient; OR, Odds ratio; 95% CI, 95% confidence interval; freq means the frequencies of allele. P values < 0.05 were marked in bold.

Supplemental Table S5. Blood pressure parameters among different genotypes in Jiangsu population.

SNP	BP parameters	Rare homozygote	Heterozygote	Frequent homozygote	p
rs16823124	SBP	144.40 ± 22.28	145.00 ± 22.42	145.00 ± 22.57	0.941
	DBP	88.04 ± 13.95	87.92 ± 12.96	86.85 ± 13.90	0.269
	PP	56.40 ± 14.58	57.12 ± 14.62	58.14 ± 14.82	0.151
	MAP	106.80 ± 15.75	107.00 ± 15.23	106.20 ± 15.81	0.672
rs2158394	SBP	145.00 ± 22.31	144.90 ± 22.66	144.90 ± 22.23	0.973
	DBP	87.87 ± 13.76	87.48 ± 13.65	87.65 ± 13.20	0.904
	PP	57.08 ± 14.39	57.44 ± 14.69	57.29 ± 14.75	0.981
	MAP	106.90 ± 15.69	106.60 ± 15.73	106.80 ± 15.25	0.949
rs1570105	SBP	145.10 ± 22.67	144.10 ± 22.15	146.10 ± 22.70	0.146
	DBP	87.69 ± 13.32	87.08 ± 13.56	88.30 ± 13.39	0.244
	PP	57.40 ± 14.80	56.99 ± 14.59	57.78 ± 14.77	0.305
	MAP	106.80 ± 15.52	106.10 ± 15.46	107.60 ± 15.81	0.160
rs2014408	SBP	142.50 ± 20.52	144.80 ± 22.05	145.40 ± 23.02	0.347
	DBP	85.51 ± 12.96	87.98 ± 13.51	87.60 ± 13.49	0.170
	PP	56.98 ± 13.63	56.86 ± 14.23	57.76 ± 15.19	0.793
	MAP	104.50 ± 14.53	106.90 ± 15.45	106.90 ± 15.7	0.200
rs7297416	SBP	145.60 ± 22.50	145.20 ± 22.74	144.30 ± 22.00	0.632
	DBP	87.50 ± 14.26	87.75 ± 13.26	87.41 ± 13.46	0.843
	PP	58.08 ± 14.66	57.43 ± 14.83	56.90 ± 14.48	0.518
	MAP	106.90 ± 16.02	106.90 ± 15.51	106.40 ± 15.35	0.779
rs59251428	SBP	144.00 ± 22.69	145.40 ± 22.60	144.60 ± 22.28	0.512
	DBP	86.41 ± 13.67	88.03 ± 13.46	87.40 ± 13.44	0.397
	PP	57.63 ± 14.46	57.41 ± 15.20	57.22 ± 14.29	0.827
	MAP	105.60 ± 15.80	107.20 ± 15.48	106.5 ± 15.51	0.422
rs3858313	SBP	145.00 ± 22.84	144.80 ± 22.88	145.00 ± 21.80	0.994
	DBP	87.50 ± 13.30	87.71 ± 13.60	87.50 ± 13.38	0.969
	PP	57.48 ± 14.90	57.14 ± 14.82	57.48 ± 14.46	0.870
	MAP	106.70 ± 15.58	106.80 ± 15.78	106.70 ± 15.21	0.988
rs4746172	SBP	144.00 ± 21.66	145.60 ± 22.79	145.60 ± 22.79	0.464
	DBP	87.47 ± 13.28	87.55 ± 13.26	87.55 ± 13.26	0.996
	PP	56.51 ± 14.26	58.01 ± 14.89	58.01 ± 14.89	0.151
	MAP	106.30 ± 15.13	106.90 ± 15.53	106.90 ± 15.53	0.839

BP, blood pressure; SBP, systolic blood pressure; DBP, diastolic blood pressure; PP, pulse pressure; MAP, median arterial pressure. All quantitative data were presented as mean ± SD. The difference of blood pressure parameters (SBP, DBP, PP, and MAP) among the genotypes of SNPs was analyzed by ANOVA adjusted age and sex.

Supplemental Table S6. Blood pressure parameters among different genotypes of the rs330693 and rs4245739 in Jiangsu population.

SNP	Genotype	SBP	DBP	PP	MAP
rs33063	AA + GA	144.90 ± 22.77	87.44 ± 13.15	57.46 ± 14.72	106.60 ± 15.49
	GG	144.90 ± 22.39	87.61 ± 13.51	57.31 ± 14.68	106.70 ± 15.52
	p	0.989	0.853	0.884	0.911
rs4245739	AC + CC	144.20 ± 21.04	86.98 ± 12.39	57.20 ± 13.36	106.00 ± 14.50
	AA	145.00 ± 22.51	87.62 ± 13.52	57.33 ± 14.75	106.70 ± 15.57
	p	0.738	0.635	0.931	0.665

BP, blood pressure; SBP, systolic blood pressure; DBP, diastolic blood pressure; PP, pulse pressure; MAP, median arterial pressure. All quantitative data were presented as mean ± SD. The difference of blood pressure parameters (SBP, DBP, PP, and MAP) between the genotypes of SNPs was analyzed by student's t-test. Number of subjects with AA and CC genotype for the rs330693 and rs4245739 were too small to analyze, so we combined AA with GA, and AC with CC, compared to GG and AA, respectively.

Supplemental Table S7. Blood pressure parameters among different genotypes in Guangdong population.

SNP	BP parameters	Rare homozygote	Heterozygote	Frequent homozygote	p
rs16823124	SBP	128.10 ± 17.55	129.50 ± 20.03	128.50 ± 18.30	0.727
	DBP	86.52 ± 11.57	84.42 ± 11.98	85.16 ± 11.65	0.436
	PP	41.60 ± 11.22	45.05 ± 14.25	43.39 ± 13.60	0.078
	MAP	100.40 ± 12.81	99.44 ± 13.58	99.62 ± 12.69	0.872
rs2158394	SBP	132.30 ± 19.47	128.2 ± 18.33	128.40 ± 19.73	0.114
	DBP	86.87 ± 9.77	84.41 ± 12.12	85.00 ± 12.08	0.139
	PP	45.41 ± 15.03	43.79 ± 12.35	43.45 ± 14.46	0.587
	MAP	102.00 ± 11.83	99.00 ± 13.27	99.48 ± 13.44	0.117
rs1570105	SBP	130.00 ± 17.89	128.5 ± 19.01	128.90 ± 19.75	0.630
	DBP	85.00 ± 10.95	85.06 ± 11.98	85.00 ± 12.07	0.971
	PP	44.96 ± 15.71	43.45 ± 12.02	43.92 ± 14.58	0.672
	MAP	99.99 ± 11.48	99.55 ± 13.57	99.64 ± 13.41	0.864
rs2014408	SBP	130.70 ± 23.56	128.10 ± 18.14	129.30 ± 19.18	0.315
	DBP	87.37 ± 10.78	83.95 ± 11.71	85.60 ± 11.93	0.140
	PP	43.37 ± 17.57	44.17 ± 13.07	43.73 ± 13.57	0.950
	MAP	101.80 ± 13.92	98.67 ± 12.77	100.20 ± 13.29	0.160
rs7297416	SBP	129.80 ± 16.71	127.80 ± 18.32	130.10 ± 20.76	0.395
	DBP	85.91 ± 11.15	84.91 ± 12.14	84.85 ± 11.62	0.669
	PP	43.92 ± 11.76	42.90 ± 12.66	45.26 ± 15.39	0.343
	MAP	100.60 ± 12.06	99.21 ± 13.21	99.93 ± 13.46	0.589
rs59251428	SBP	128.50 ± 19.72	128.30 ± 19.33	129.50 ± 18.61	0.629
	DBP	83.87 ± 11.69	84.93 ± 11.90	85.39 ± 11.76	0.634
	PP	44.60 ± 15.02	43.41 ± 12.86	44.15 ± 13.93	0.773
	MAP	98.73 ± 13.06	99.41 ± 13.50	100.1 ± 12.83	0.609
rs3858313	SBP	127.60 ± 18.21	128.80 ± 17.99	129.50 ± 20.38	0.883
	DBP	84.47 ± 11.74	85.63 ± 11.18	84.63 ± 12.47	0.397
	PP	43.16 ± 12.15	43.22 ± 12.98	44.89 ± 14.73	0.672
	MAP	98.86 ± 13.03	100.00 ± 12.40	99.59 ± 13.92	0.654
rs4746172	SBP	130.30 ± 18.72	128.30 ± 20.21	129.20 ± 17.89	0.559
	DBP	86.76 ± 11.99	83.74 ± 12.23	85.88 ± 11.19	**0.020**
	PP	43.56 ± 12.12	44.61 ± 13.9	43.32 ± 13.65	0.417
	MAP	101.30 ± 13.41	98.61 ± 13.89	100.30 ± 12.20	0.110

BP, blood pressure; SBP, systolic blood pressure; DBP, diastolic blood pressure; PP, pulse pressure; MAP, median arterial pressure. All quantitative data were presented as mean ± SD. The difference of blood pressure traits (SBP, DBP, PP, and MAP) among the genotypes of SNPs was analyzed by ANOVA adjusted age and sex. P values < 0.05 were marked in bold.

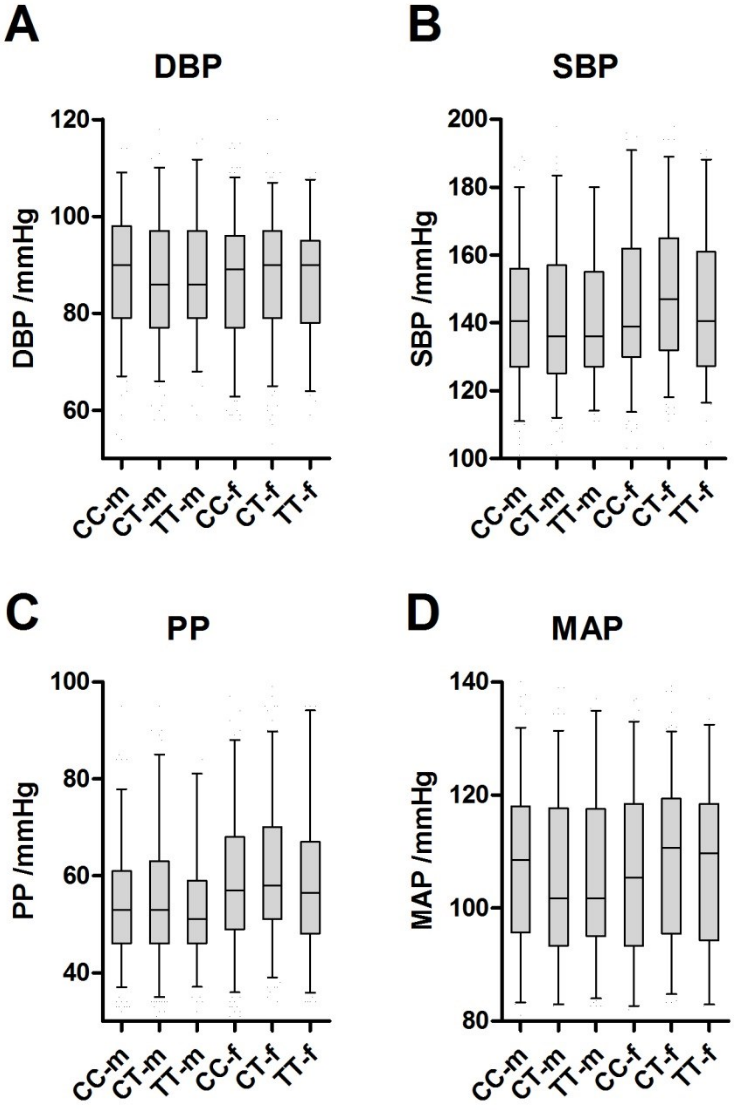

Supplemental Figure S1. Blood parameters among different genotypes of the rs4746172 in male and female subjects of Jiangsu population. DBP, diastolic blood pressure; SBP, systolic blood pressure; PP, pulse pressure; MAP, median arterial pressure; CC, CT and TT are the genotypes of rs4746172; f and m mean female and male, respectively. The values of BP parameters were presented as median, 5-95 percentile.
